# Developing citizen report cards for primary health care in low and middle-income countries: Results from cognitive interviews in rural Tajikistan

**DOI:** 10.1371/journal.pone.0186745

**Published:** 2017-10-24

**Authors:** Sebastian Bauhoff, Lila Rabinovich, Lauren A. Mayer

**Affiliations:** 1 Center for Global Development, Washington, District of Columbia, United States of America; 2 Center for Economic and Social Research, University of Southern California, Arlington, Virginia, United States of America; 3 RAND Corporation, Pittsburgh, Pennsylvania, United States of America; National Institute of Health, ITALY

## Abstract

Citizen report cards on health care providers have been identified as a potential means to increase citizen engagement, provider accountability and health systems performance. Research in high-income settings indicates that the wording, presentation and display of performance information are critical to achieve these goals. However, there are limited insights on developing effective report card designs for middle- and low-income settings. We conducted cognitive interviews to assess consumers’ understanding, interpretation of and preferences for displaying information for a health care report card in rural Tajikistan. We recruited a convenience sample of 40 citizens (20 women and 20 men aged 18–45) from rural areas of two provinces of Tajikistan (Soghd and Khatlon *oblasts)*. The interview protocol was adapted from the model of cognitive interviews used in social science research to improve survey questionnaires. We used multivariate regression to assess understanding and interpretation of the report card; chi2 tests to assess differences in preferences for displaying information; and tests of proportions to assess the preferred comparison group. Respondents understood the main idea of the report card and are not confused by the indicators or display. However, many respondents had difficulties making comparisons, and when asked to identify worst-performing services. Respondents preferred detailed rankings using school grades, comparisons of their local clinic with the regional or national average performance, and the use of color in the report card. We found some heterogeneity across the two provinces. Overall, our findings are promising regarding the citizens’ comprehension of health care report cards in rural Tajikistan, while underscoring the challenges of effectively providing health care performance information to communities. Cognitive interviews and iterative testing can support an effective implementation of reporting initiatives.

## Introduction

Public report cards are one tool in a wider suite of approaches to increase the accountability and performance of service providers, particularly in the healthcare field [1–6]. Report cards, variously called consumer reports, score cards, citizen report cards and quality assessment reports [7], are primarily targeted to consumers and compare the performance of specific providers along a number of indicators. Most of the existing research on the use of healthcare quality report cards originates in high-income countries, primarily the United States. In such settings, report cards are often intended to inform the choice of provider (e.g., [1]) and may serve to complement regulatory approaches to maintain performance and quality standards [6]. In contrast, report card initiatives in low- and middle-income countries tend to focus on facilitating citizen engagement and priority setting by introducing monitoring and accountability to the public sector [4,8]. Research in a range of low-income settings suggests that report cards can improve provider accountability, for instance, by improving local community oversight and engagement which can motivate providers and lead to joint action to address larger problems. [9–12]. The effectiveness of these initiatives in improving quality of care in low-income settings, however, has not yet been firmly established.

While a number of developing countries have employed some form of a report card (e.g., [2–4,10,11]), little is known about these consumers’ understanding of the aims and content of report cards, or about their preferences for report card presentation [4,13]. Exploring these areas is important, as report cards could have potentially high impact in improving service provision and quality in resource-constrained settings [7,13,14].

Current research in low- and middle-income countries provides guidance on the fundamental steps for determining the content and implementation of report cards [7,15]. However, there is limited insight into determining what wording, presentation and display ensure that the report cards’ information is most suitable to the target audience. Indeed, research on healthcare report cards in the United States suggests that even small modifications can have large effects on consumer perceptions and comprehension, as well as usefulness and relevance (e.g., [16–19]).

Research in communication and cognitive psychology indicates that consumers are in principle able to understand and engage in in-depth discussions on complex topics, including information presented in report card style that characterizes a set of choices and systematically compares them along a set of attributes [20–23]. However, studies on high-income settings also find that consumers may have difficulty understanding and accessing the information presented to them (e.g., [1,24]) and that the complexity of healthcare quality report cards affects how consumers process the information [17]. This has led to detailed recommendations for designing report cards on health care providers and health insurers [25–28], often with the goal of facilitating choice. Issues of understanding and interpretation also likely apply to report cards aiming to enhance the accountability of providers in low- and middle-income countries. In either setting, testing and revising report cards is a key step to improving the effectiveness of report cards and their relevance to the target audience.

We conducted cognitive interviews in two Tajik provinces (Soghd and Khatlon *oblasts*) to test consumers’ understanding and interpretation, as well as display preferences, of a report card developed specifically for primary health care providers in rural Tajikistan.

### Policy context

In Tajikistan, health care facilities are almost exclusively state-owned and controlled by the Ministry of Health, whereas health financing is decentralized [29]. Like other countries in the region, the health system struggles simultaneously with a high burden of chronic diseases and an under-performing primary health care system [30].

Tajikistan is considering the deployment of public report cards to improve the performance and accountability of primary health care providers, and to enable communities to participate in priority setting for local service provision by providing information necessary to advocate for improvements. Unlike other settings in which report cards have been utilized, consumers in rural Tajikistan tend to have little, if any, choice in healthcare service provider, with typically only one public primary and secondary provider serving rural Tajik communities, and a lack of (affordable) private alternatives [29].

As a result, the primary objectives of a public reporting initiative in rural Tajikistan are to track performance and increase accountability rather than to inform choice of providers. Medical services are often unavailable and unofficial payments are highly prevalent; formal grievance mechanisms remain mostly unused, as few citizens know where to file a complaint [31]. The Tajik government recognized the potential role of community involvement in prioritizing improvements in the health care system and monitoring providers [30].

### Development of the prototype report card

This study took place in the context of a larger effort to improve health systems performance by the Tajik government, and partly supported by the World Bank. The goal of the study was to develop a draft citizen report card that is comprehensible and relevant to laypersons in rural Tajikistan. The report card focused on two critical clinical domains of care in this setting, maternal and child health (MCH) and cardiovascular diseases and diabetes (CVDD), and contained a number of indicators for each domain. The report card also included indicators for overall facility conditions. We used a sequential approach to develop the final design. In an initial phase, we conducted a desk review of research on health care performance reporting, complemented with formative qualitative research in rural Tajikistan. While not specific to the Tajik context, existing literature provides important lessons for the development of report cards on provider performance in Tajikistan. In particular, existing literature highlights the need to minimize the cognitive capacity needed to correctly interpret the report card information [32,33], as well as visual design considerations to communicate the intended message efficiently and effectively while engaging readers [34]. Practical advice from the existing literature includes using general categories of information that are relevant to users; using a clear and consistent structure to convey the information; and using a employing a structure that facilitates quick access to information of interest, e.g., using columns to display comparisons [28].

Findings from formative qualitative research (focus groups) with members of the target audience in rural Tajikistan yielded several specific insights for the development of the prototype report card. First, the majority of participants indicated a preference for numerical values or words rather than symbols, such as smiley faces or stars, to present the information. Second, the preferred numerical scoring is the school grading system (i.e., a scoring from 1 to 5), a ranking that is commonly understood in rural Tajikistan. Third, while participants tended to agree on the importance of certain indicators of healthcare performance (such as healthcare personnel responsiveness and clinical management), men and women ranked the relative importance of other indicators (such as availability of obstetrical equipment) very differently. This suggests the need to carefully consider the types of indicators included in a report card, and the overall balance between them, to ensure relevance to all members of the intended audience.

**Fig 1:**
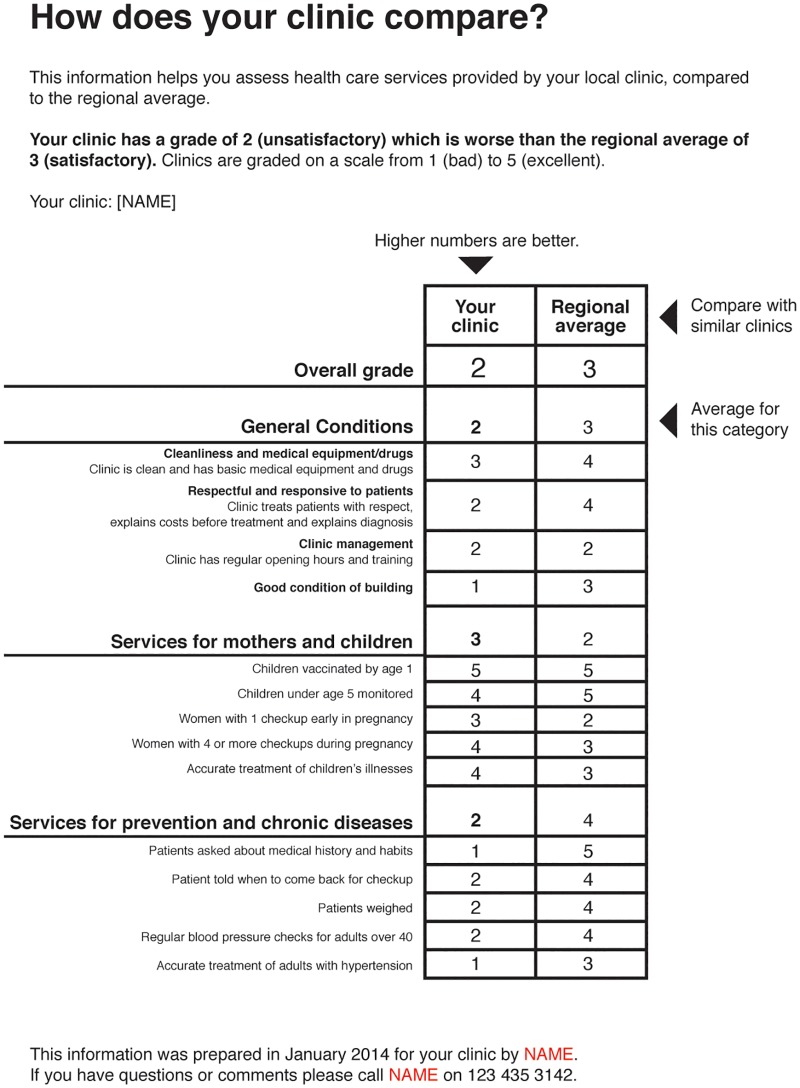
Prototype report card.

We used these initial insights to develop a prototype report card (Fig 1) to test using cognitive interviews to evaluate citizen’s understanding and interpretation, as well as their preferences in terms of report card wording, presentation and display. The prototype was designed to draw attention and engage the reader, starting with the title (“How does your clinic compare?”). The graphical design aims to avoid visual distractions and provide cues to guide the reader. The report card uses simple language and avoids complex words that are difficult to understand or interpret, as suggested by Hibbard et al (2010). For example, we avoided the term “maternal and child health” throughout and instead used “services for mothers and children”. We added wording to illustrate how the information should be interpreted. For instance, the header of the report card includes a sentence “Your clinic has a grade of 2 (unsatisfactory) which is worse than the regional average of 3 (satisfactory)”. We provided similar descriptions below some of the more complex indicators; for instance, for the indicator “Respectful and responsive to patients” we added a basic explanation: “Clinic treats patients with respect, explains costs before treatment, and explains diagnosis”. The report card also clarifies the scoring scheme used to indicate the quality of a provider’s service in particular areas; following the findings from the formative research we used a numerical scheme based on school grades and explained their meaning in the instructions to readers (“Clinics are graded on a scale from 1 (bad) to 5 (excellent)”).

The prototype report card focused on two specific domains of care (MCH and CVDD), and also captured general facility infrastructure, supplies and management. We used these three categories to group indicators. The indicators for the prototype were selected to be illustrative of a range of issues (e.g., accessibility, cost, provider responsiveness) and varying degrees of complexity.

## Materials and methods

We tested the report card prototype with members of the intended target audience using a cognitive interview technique, an approach also used with U.S. consumers [24]. In this section, we describe the sample and methodological approach for this testing. Free and informed consent was obtained from all respondents. Verbal consent was used in light of low literacy levels in rural Tajikistan, and recorded by interviewers. This approach and the overall research was approved by the RAND Corporation Human Subjects Protection Committee (study number 2013–0227).

### Sample and recruitment

We conducted 40 interviews to assess citizens’ understanding and interpretation, as well as their preferences regarding the presentation of the content. A convenience sample of respondents was recruited from Khatlon and Soghd provinces, with interviews evenly split between the two. Respondents were sampled from the general rural population in two districts of each province, and the sample was stratified by gender (with half of the sample in each province being female) and age (half the sample were between 18 and 45, and the other half over 45). Respondents were paired with interviewers of the same gender. S1 Table provides an overview of the sample.

### Interviewing approach

The interview protocol was adapted from the model of cognitive interviews used in social science research to improve survey questionnaires [35–38]. Broadly, cognitive interviewing is used to understand how people process information, including factors such as attention span, word recognition, memory, and language processing [36]. The process involves the administration of draft survey questions while simultaneously probing respondents to comment on their interpretation, understanding and perceptions of the questions [35]. The basic form of cognitive interviewing consists of semi-structured, in-depth interviews with members of the target population [39]. Interviews may involve ‘think-aloud’ questions, whereby respondents are asked to “provide a verbal account of their thinking” as they are presented with the survey’s questions, with interviewers probing respondents with further questions to elicit greater detail or obtain additional information [36]. Verbal probing, whereby the interviewer asks specific questions about the respondent’s survey answers is sometimes used alongside ‘think-aloud’ questions [39] and may yield more targeted and potentially more useful information from respondents in the course of a short interview [35]. Cognitive interviewing protocols can be completely scripted, somewhat scripted, or fully improvised [35]. This methodology allows researchers to identify potential pitfalls with their survey instrument prior to deployment, such as respondents understanding the questions in an inconsistent way, or the questions asking for information respondents do not have and/or cannot retrieve [37].

While used extensively to identify and address problems with questionnaires, cognitive interviewing is less often used in the development and evaluation of communications such as public health messages and materials, and then almost always in the U.S. and other high-income country settings [24,40].

We adapted cognitive interviewing to garner citizen feedback to refine the report card mockup. In our interviews, we primarily used verbal probing, asking respondents specific questions about the report card after it was shown to them. For example, the interviewer pointed to a particular section or feature of the report card and asked respondents to describe it in their own words. We also provided alternative versions of certain sections of the report card to explore respondents’ preferences regarding the report cards’ design and content. We focused on respondents’ understanding and interpretation of the report card, and preferences for displaying the information and for including comparisons (see S1 Fig for the displays).

First, we tested respondents’ “understanding” of the purpose and content of the report card by asking them to explain the overall purpose of the report card and the meaning of various terms in their own words. For instance, we asked respondents to explain the meaning of “services for mothers and children” and provide examples of relevant provider activities.

Second, we tested respondents’ “interpretation” by asking them to interpret the information on the report card in their own words. For instance, we asked respondents to identify the best/worst performing service, either within-clinic or in comparison to the regional average.

Third, we assessed respondent “preferences for displaying information" with regards to the level of detail, scale of the ratings (school grades, percent or verbal), and use of color and graphics. We elicited these responses after showing respondents variations of our initial mockup that altered these attributes one at a time. For instance, we used alternative versions of sections of the mockup to ask respondents about their preference for color-coding and the level of detail in the report card.

Fourth, we asked respondents about their “preferences for comparisons” or what would be the most useful comparison for their local clinic (regional or national average; own clinic over time; or specific clinics nearby).

Finally, as a separate module in the interview, we explored respondents’ “preferences for report card deployment” or their views on effective ways for the deployment of the report card in the community so as to gain preliminary insights for a possible reporting initiative in Tajikistan.

The protocols were initially prepared in English and then translated into Tajik. Tajik protocols were piloted in the districts of Yavan (Khatlon province) and D. Rasulov (Soghd province). Each interview lasted between 30 and 60 minutes, and was recorded in full for subsequent transcription. Interviews were conducted in Tajik or Uzbek, depending on the area. Recordings were transcribed and translated into English. The protocols were entered into a database using predefined answer codes that interviewers could select; some answer codes were adjusted based on the text responses in the transcripts. We obtained consent from respondents to conduct and record the interview. Each participant was offered compensation equivalent to USD 5 in local currency.

### Analysis

We used multivariate linear regressions to evaluate the associations of respondent characteristics with understanding and interpretation of the report cards. The respondent characteristics were captured by indicator variables for gender, province (Khatlon and Soghd) and age groups (aged 18–46 and 46 and older). We constructed summary indices for the dependent variables so as to combine 10 questions for “understanding” and 7 questions for “interpretation”. The two indices are the average of binary variables that indicate whether a response was correct, with ranges from 0 (no correct answer) to 100 (all answers correct).

We used chi2 tests to assess similarities in the distributions of categorical responses regarding preferences for displaying the information across the two provinces, Soghd and Khatlon. We used two-sided tests of proportions to assess whether preferences for various possible comparisons differed across regions. We focused on the regional comparison since, in practice, the report cards could be adapted to each locality.

We coded and tabulated the responses for the exploratory research on preferences for the deployment of the report cards.

## Results

### Understanding and interpretation of the report card

Table 1 shows the results from the linear regression regarding consumers’ understanding and interpretation of the report card. The sample mean is included in the table footer.

**Table 1:** Multivariate regressions of the understanding and interpretation indices (percentage point changes).

	Understanding index	Interpretation index
Male	-3.1	-7.8
	(3.7)	(7.4)
Age 18–45	2.1	0.5
	(3.7)	(7.4)
Khatlon province	9.8**	-14.5*
	(3.7)	(7.4)
Constant	76.8***	67.3***
	(4.0)	(5.9)
Sample mean	81.1	56.3
R-squared	0.19	0.12
N	40	40

* p<0.10,

** p<0.05,

*** p<0.01.

Indices scaled 0 (no question answered correctly) to 100 (all questions answered correctly). OLS regressions with robust standard errors in parenthesis. Omitted categories are: female; age 46+; Soghd province. Dependent variables are unweighted averages of 10 (understanding) and 7 (interpretation) questions.

The sample-average score of 56% for the “understanding” index indicates that a large number of respondents had difficulty explaining the meaning of the report card content or provide examples for the categories and services listed on the report card, such as “maternal and child health” and “children under age 5 monitored”. Respondents in Khatlon had a statistically significantly higher score than respondents in Soghd. We separately examined one subcomponent of this index, whether respondents could explain the main idea or message of the report card in their own words (results not shown). 87% of respondents were able to do so, with responses such as “to know more about the policlinic’s services”, “[to learn] what kind of progress [was made] in the last years”, and “to assess the policlinic”.

The average score for the “interpretation” index is 81%, which suggests that some respondents struggle to interpret the relative ratings of different services. On closer inspection, respondents were better at identifying services that were better-performing, either within-clinic or in comparison with the regional average, than at identifying the worst-performing service. This is puzzling since the cognitive task for these two comparisons is fundamentally similar. The finding could reflect a reluctance to single out poor performance, perhaps shaped by cultural mores. There is also some suggestive evidence that respondents may be better at comparing ratings within a column (comparing services within the local clinic) than across columns (comparing ratings between the local clinic and the regional average). While we do not have a definitive explanation for this finding, it is possible that people find the task of comparing the performance of their local clinic to the average performance of all regional clinics cognitively complex, particularly in the somewhat stressful situation of an interview.

### Preferences for displaying information

Table 2 shows the expressed preferences for level of detail and scale of ratings, and the use of colors and graphics. When presented with alternative versions of the report card containing different levels of textual detail and explanation, respondents preferred the level of detail in the original mockup, i.e., category headings with the names of indicators and a short description rather than merely category headings and/or indicator names. Overall, respondents weakly preferred the school grade scale used in the mockup (from 1 (bad) to 5 (excellent)), but some respondents also expressed interest in seeing the ratings in words. Although the distribution of responses in Soghd and Khatlon was significantly different, respondents in both locations gave the school grades the highest preference. When asked directly what they found attractive in the presentation, most respondents mentioned the table format used in the mockup; they also thought that the amount of information and the text size were about right (results not shown).

**Table 2:** Preferences for displaying information (count and column percent).

	Soghd		Khatlon	
Detail (p = 0.35)	N	(%)	N	(%)
A lot of detail	18	(95)	15	(75)
Some detail	1	(5)	3	(15)
Little detail	0	(0)	1	(5)
No preference; don't know	0	(0)	1	(5)
**Rating scale** (p = 0.08)				
School grades only	10	(50)	8	(40)
Percent only	6	(30)	6	(30)
Words only	1	(5)	6	(30)
No preference; don't know	3	(15)	0	(0)
**Color** (p = 0.51)				
No color	3	(15)	1	(5)
Color	16	(80)	17	(85)
No preference; don't know	1	(5)	2	(10)
**Graphics** (p = 0.67)				
Original card (no boxes or pictures)	3	(15)	2	(10)
Original card with boxes	2	(10)	1	(5)
Original card with pictures	15	(75)	16	(80)
No preference; don't know	0	(0)	1	(5)

P-value based on Pearson's chi2 test. Preferences elicited after showing respective mockups to respondent; each display attribute was shown separately and relative to baseline condition (listed in the first rows). Overall sample size N = 40; counts may vary due to item non-response.

Respondents also indicated a preference for report cards with colors and pictures over report cards in black and white and with only letters and numbers. This finding may reflect a preference for report cards that are attractive and draw attention, a suggestion also expressed by several respondents and interviewers. When asked directly how the presentation could be improved most respondents preferred graphics and pictures over colors. It is not clear, however, whether the colors and pictures aid with textual comprehension, help signpost different sections, or merely add visual interest and appeal.

### Preferences for comparisons

Table 3 suggests that despite the difficulties in making comparisons, respondents view including comparisons as useful, with a majority preferring the comparison with the regional average over the comparisons with either a named clinic in a neighboring region or with the national average. However, while respondents Khatlon stated that most of the possible comparisons would be useful, those in Soghd strongly favored the regional average, with 79% of respondents compared to 44% in Khatlon.

**Table 3:** Most useful comparisons with local clinic (proportions).

	Soghd	Khatlon	p-value
Regional average	0.79	0.44	0.03
National average	0.00	0.50	0.00
Own clinic over time	0.16	0.28	0.38
Specific clinics	0.11	0.28	0.18

Multiple answers allowed. P-value from two-sided test of proportions. 19 and 18 observations in Soghd and Khatlon respectively.

### Preferences for report card deployment

Responses to our formative questions on report card deployment in the community suggested almost unequivocally that it would be important to explain the report card to citizens when it is first distributed (results not shown). Some respondents suggested holding local meetings to explain the report cards, while others suggested involving local community leaders in disseminating the information.

Based on the above findings we revised the report card prototype used in this study (see S2 Fig).

## Discussion

Accountability interventions have been identified as potential means to increase citizen engagement and improve the performance of service providers [8]. Public report cards are one tool for increasing accountability in resource-constrained settings [1,5]. Although there is significant evidence from high-income settings that the presentation of the information is crucial to a reporting effort, there is limited guidance as to how to design these kinds of communication materials for citizens in low- and middle-income countries, a particularly challenging environment. As the interest and popularity of report cards increases in developing country contexts, testing and refining their design should be a priority.

In this paper we discuss findings from cognitive interviews of a prototype report card in two provinces of rural Tajikistan. The prototype was developed on the basis of prior formative qualitative research with the target population and a review of best practice literature on communications in health care quality and performance; the cognitive interviews were used to evaluate understanding and interpretation of the report card’s content, as well as preferences for displaying the information.

Our findings show that consumers find certain tasks easier than others. Specifically, we found that respondents understand the main idea of the report card and are not confused by the indicators or display. However, we also found that respondents in our study sample had difficulties making comparisons, especially between the hypothetical local clinic and the regional average, and when asked to identify worst-performing services. Respondents’ were for the most part unable to retrieve the information in the report card about the comparative quality of the two providers. Moreover, most respondents focused primarily on the information about their own clinic, partially or completely disregarding the comparison provided in the report card. This might suggest a number of possible avenues to modify the report card. One option could be to start with the comparison against the regional average at baseline and, once a second wave of performance data is available, switching to a comparison of the same local clinic over time. A comparison of the performance of consumers’ own clinic over time might appeal to their focus on their local provider. In our study, respondents in Soghd and Khatlon preferred the regional and national comparison respectively. Our findings also indicate possible heterogeneities by gender, which may suggest the need to tailor dissemination and engagement activities within a locality.

These findings resonate with those from the literature in high-income settings. The overall cognitive burden of report cards may be high and can lower motivation to engage with the information, and lead to misunderstandings. Comparisons are particularly challenging to consumers in these settings and in our study population, suggesting that report cards need to offer substantial assistance for consumers to access and interpret the data (e.g., [25]). This could explain our findings of a preference for the school grade scale (an existing and understood approach to rating) and short descriptions (in effect, help with interpretation).

Overall, our findings highlight the need to test report cards (and other communication materials) not just for indicator preference and comprehension, but also against consumers’ ability to contextualize the information and relate individual pieces of information to other relevant ones in the report card. In addition, our findings indicate that cognitive interviewing techniques previously used in high-income settings can be used to identify critical parameters for designing report cards and other communications materials in low-income countries. Cognitive interviewing may hold promise as a method to develop, assess and improve communications in health care and other fields in middle- and low-income settings, as it can help practitioners (such as governments, NGOs, donors) to better tailor communication, education and other materials.

This study has several limitations. Cognitive interviewing can be demanding on the respondent, which imposes constraints on the breadth and depth of the exercise. While we were able to test some variations to the mock report card and significant issues of word-choice, substance and presentation, it was not possible to test entire alternative report cards, thus limiting our insights into preferred layout, optimal language and most appropriate content. In addition, cognitive interviewing does not provide insights into the best ways to disseminate the report card, a crucial aspect of the report card approach to increased provider accountability. Moreover, our study sample is limited in number and diversity, and our findings may not be representative of the larger Tajik population. Finally, communication design is an inherently iterative process and there remains scope for further testing and refining the report card. Our protocol captured the several dimensions of understanding, interpretation and presentation, but did not consider in detail the interaction of design elements; further improvements in wording may also be warranted. Additional testing could help identify additional ways to facilitate comprehension and identify a more nuanced set of visual aids (pictures, color) for the report card. Similarly, our study does not explicitly consider other elements that are important to the successful implementation of report cards in this setting, such as involving communities in setting priorities for reporting and improvement or engaging citizens to use the report cards for monitoring local providers. It will be useful to monitor comprehension and the use of the information as the report card is initially implemented and subsequently updated.

## Conclusions

Overall, our findings are promising regarding the citizens’ comprehension of health care report cards in rural Tajikistan, while underscoring the challenges of effectively providing health care performance information to communities. Importantly, our study focused on understanding and interpretation as two critical elements of successful reporting initiatives. Future research on designing effective report cars and other communication tools in low-resource settings could examine approaches to addressing heterogeneity across populations and stakeholders (e.g. consumers and providers) in public accountability and consumer information initiatives. Moreover, initiatives that aim to increase community involvement in priority setting or monitoring health care providers may also need to develop effective ways to engage citizens beyond providing report cards.

## Supporting information

S1 Fig Alternative versions of the report cards shown to respondents. (PDF)

S2 Fig Revised report card template. (PDF)

S1 Table Sample for cognitive interviews (counts). (PDF)
