# Supplementary material for: Developing citizen report cards for primary health care in low and middle-income countries: Results from cognitive interviews in rural Tajikistan
Source: PLoS One. 2017 Oct 24;12(10):e0186745. doi: 10.1371/journal.pone.0186745 (PMC5655492; doi:10.1371/journal.pone.0186745)
Supplement: S1 Fig — (PDF) [file pone.0186745.s001.pdf]

**S1 Fig. Alternative versions of the report cards shown to respondents.**

## Presentation: Detail

### A

| | **Your clinic** | Regional average |
|---|---|---|
| **Overall grade** | **2** | 3 |
| **General conditions** | **2** | 3 |
| **Cleanliness and medical equipment/drugs**<br>Clinic is clean and has basic medical equipment and drugs | 3 | 4 |
| **Respectful and responsive to patients**<br>Clinic treats patients with respect,<br>explains costs before treatment and explains diagnosis | 2 | 4 |
| **Clinic management**<br>Clinic has regular opening hours and training | 2 | 2 |
| **Good condition of building** | 1 | 3 |

### B

| | **Your clinic** | Regional average |
|---|---|---|
| **Overall grade** | **2** | 3 |
| **General conditions** | **2** | 3 |
| Cleanliness and medical equipment/drugs | 3 | 4 |
| Respectful and responsive to patients | 2 | 4 |
| Clinic management | 2 | 2 |
| Good condition of infrastructure | 1 | 3 |

### C

| | **Your clinic** | Regional average |
|---|---|---|
| **Overall grade** | **2** | 3 |
| General conditions | 2 | 3 |
| Services for mothers and children | 3 | 2 |
| Services for prevention and chronic diseases | 2 | 4 |

## Presentation: Scale

**A** Clinics are graded on a scale from 1 (bad) to 5 (excellent).

| | Your clinic | Regional average |
|---|---|---|
| **Overall grade** | **2** | 3 |
| **General conditions** | **2** | 3 |
| **Cleanliness and medical equipment/drugs**<br>Clinic is clean and has basic medical equipment and drugs | 3 | 4 |
| **Respectful and responsive to patients**<br>Clinic treats patients with respect, explains costs before treatment and explains diagnosis | 2 | 4 |
| **Clinic management**<br>Clinic has regular opening hours and training | 2 | 2 |
| **Good condition of building** | 1 | 3 |

**B** Clinics are graded on a scale from 0 (bad) to 100 (excellent).

| | Your clinic | Regional average |
|---|---|---|
| **Overall grade** | **32%** | 48% |
| **General conditions** | **29%** | 50% |
| **Cleanliness and medical equipment/drugs**<br>Clinic is clean and has basic medical equipment and drugs | 46% | 67% |
| **Respectful and responsive to patients**<br>Clinic treats patients with respect, explains costs before treatment and explains diagnosis | 21% | 71% |
| **Clinic management**<br>Clinic has regular opening hours and training | 28% | 22% |
| **Good condition of infrastructure** | 19% | 48% |

**C** Clinics are graded on a scale from bad to excellent.

| | Your clinic | Regional average |
|---|---|---|
| **Overall grade** | **Unsatisfactory** | Satisfactory |
| **General conditions** | **Unsatisfactory** | Satisfactory |
| **Cleanliness and medical equipment/drugs**<br>Clinic is clean and has basic medical equipment and drugs | Satisfactory | Good |
| **Respectful and responsive to patients**<br>Clinic treats patients with respect, explains costs before treatment and explains diagnosis | Unsatisfactory | Good |
| **Clinic management**<br>Clinic has regular opening hours and training | Unsatisfactory | Unsatisfactory |
| **Good condition of building** | Bad | Satisfactory |

## Presentation: Comparison

### A

| | Your clinic | Regional average |
|---|---|---|
| **Overall grade** | **2** | 3 |
| | | |
| **General conditions** | **2** | 3 |
| **Cleanliness and medical equipment/drugs**<br>Clinic is clean and has basic medical equipment and drugs | 3 | 4 |
| **Respectful and responsive to patients**<br>Clinic treats patients with respect, explains costs before treatment and explains diagnosis | 2 | 4 |
| **Clinic management**<br>Clinic has regular opening hours and training | 2 | 2 |
| **Good condition of building** | 1 | 3 |

### B

| | Your clinic |
|---|---|
| **Overall grade** | **2** |
| | |
| **General conditions** | **2** |
| **Cleanliness and medical equipment/drugs**<br>Clinic is clean and has basic medical equipment and drugs | 3 |
| **Respectful and responsive to patients**<br>Clinic treats patients with respect, explains costs before treatment and explains diagnosis | 2 |
| **Clinic management**<br>Clinic has regular opening hours and training | 2 |
| **Good condition of building** | 1 |

### C

| | Your clinic this year | Your clinic last year |
|---|---|---|
| **Overall grade** | **2** | 3 |
| | | |
| **General conditions** | **2** | 3 |
| **Cleanliness and medical equipment/drugs**<br>Clinic is clean and has basic medical equipment and drugs | 3 | 4 |
| **Respectful and responsive to patients**<br>Clinic treats patients with respect, explains costs before treatment and explains diagnosis | 2 | 4 |
| **Clinic management**<br>Clinic has regular opening hours and training | 2 | 2 |
| **Good condition of building** | 1 | 3 |

# Presentation: Graphical display

Higher numbers are better.

## A

| | Your clinic | Regional average |
|---|---|---|
| **<u>Overall grade</u>** | **<u>2</u>** | <u>3</u> |
| **General conditions** | **2** | 3 |
| **Cleanliness and medical equipment/drugs**<br>Clinic is clean and has basic medical equipment and drugs | 3 | 4 |
| **Respectful and responsive to patients**<br>Clinic treats patients with respect,<br>explains costs before treatment and explains diagnosis | 2 | 4 |
| **Clinic management**<br>Clinic has regular opening hours and training | 2 | 2 |
| **Good condition of building** | 1 | 3 |

Higher numbers are better.

## B

| | Your clinic | Regional average |
|---|---|---|
| **<u>Overall grade</u>** | **<u>2</u>** | <u>3</u> |
| **General conditions** | **2** | 3 |
| **Cleanliness and medical equipment/drugs**<br>Clinic is clean and has basic medical equipment and drugs | 3 | 4 |
| **Respectful and responsive to patients**<br>Clinic treats patients with respect,<br>explains costs before treatment and explains diagnosis | 2 | 4 |
| **Clinic management**<br>Clinic has regular opening hours and training | 2 | 2 |
| **Good condition of building** | 1 | 3 |

1 (bad)
2 (unsatisfactory)
3 (satisfactory)
4 (good)
5 (excellent)

**Presentation: Overall**

A

# How does your clinic compare?

This information helps you assess health care services provided by your local clinic, compared to the regional average.

**Your clinic has a grade of 2 (unsatisfactory) which is worse than the regional average of 3 (satisfactory).** Clinics are graded on a scale from 1 (bad) to 5 (excellent).

Your clinic: [NAME]

Higher numbers are better.

| | Your clinic | Regional average |
|---|---|---|
| **Overall grade** | **2** | 3 |
| **General conditions** | **2** | 3 |
| **Cleanliness and medical equipment/drugs** Clinic is clean and has basic medical equipment and drugs | 3 | 4 |
| **Respectful and responsive to patients** Clinic treats patients with respect, explains costs before treatment and explains diagnosis | 2 | 4 |
| **Clinic management** Clinic has regular opening hours and training | 2 | 2 |
| **Good condition of building** | 1 | 3 |
| **Services for mothers and children** | **3** | 2 |
| Children vaccinated by age 1 | 5 | 5 |
| Children under age 5 monitored | 4 | 5 |
| Women with 1 checkup early in pregnancy | 3 | 2 |
| Women with 4 or more checkups during pregnancy | 4 | 3 |
| Accurate treatment of children's illnesses | 4 | 3 |
| **Services for prevention and chronic diseases** | **2** | 4 |
| Patients asked about medical history and habits | 1 | 5 |
| Patient told when to come back for checkup | 2 | 4 |
| Patients weighed | 2 | 4 |
| Regular blood pressure checks for adults over 40 | 2 | 4 |
| Accurate treatment of adults with hypertension | 1 | 3 |

Compare with similar clinics

Average for this category

This information was prepared in January 2014 for your clinic by NAME.
If you have questions or comments please call NAME on 123 435 3142.

B

# How does your clinic compare?

This information helps you assess health care services provided by your local clinic, compared to the regional average.

**Your clinic has a grade of 2 (unsatisfactory) which is worse than the regional average of 3 (satisfactory).** Clinics are graded on a scale from 1 (bad) to 5 (excellent).

Your clinic: [NAME]

Higher numbers are better.

| | Your clinic | Regional average |
|---|---|---|
| **Overall grade** | **2** | 3 |

Compare with similar clinics

| **General conditions** | **2** | 3 |
|---|---|---|
| **Cleanliness and medical equipment/drugs**<br>Clinic is clean and has basic medical equipment and drugs | 3 | 4 |
| **Respectful and responsive to patients**<br>Clinic treats patients with respect, explains costs before treatment and explains diagnosis | 2 | 4 |
| **Clinic management**<br>Clinic has regular opening hours and training | 2 | 2 |
| **Good condition of building** | 1 | 3 |

| **Services for mothers and children** | **3** | 2 |
|---|---|---|
| Children vaccinated by age 1 | 5 | 5 |
| Children under age 5 monitored | 4 | 5 |
| Women with 1 checkup early in pregnancy | 3 | 2 |
| Women with 4 or more checkups during pregnancy | 4 | 3 |
| Accurate treatment of children's illnesses | 4 | 3 |

| **Services for prevention and chronic diseases** | **2** | 4 |
|---|---|---|
| Patients asked about medical history and habits | 1 | 5 |
| Patient told when to come back for checkup | 2 | 4 |
| Patients weighed | 2 | 4 |
| Regular blood pressure checks for adults over 40 | 2 | 4 |
| Accurate treatment of adults with hypertension | 1 | 3 |

This information was prepared in January 2014 for your clinic by NAME.
If you have questions or comments please call NAME on 123 435 3142.

C

# How does your clinic compare?

This information helps you assess health care services provided by your local clinic, compared to the regional average.

**Your clinic has a grade of 2 (unsatisfactory) which is worse than the regional average of 3 (satisfactory).** Clinics are graded on a scale from 1 (bad) to 5 (excellent).

Your clinic: [NAME]

Higher numbers are better.

Compare with similar clinics

Average for this category

| | Your clinic | Regional average |
|---|---|---|
| **Overall grade** | **2** | 3 |
| **General conditions** | **2** | 3 |
| **Cleanliness and medical equipment/drugs** Clinic is clean and has basic medical equipment and drugs | 3 | 4 |
| **Respectful and responsive to patients** Clinic treats patients with respect, explains costs before treatment and explains diagnosis | 2 | 4 |
| **Clinic management** Clinic has regular opening hours and training | 2 | 2 |
| **Good condition of building** | 1 | 3 |
| **Services for mothers and children** | **3** | 2 |
| Children vaccinated by age 1 | 5 | 5 |
| Children under age 5 monitored | 4 | 5 |
| Women with 1 checkup early in pregnancy | 3 | 2 |
| Women with 4 or more checkups during pregnancy | 4 | 3 |
| Accurate treatment of children's illnesses | 4 | 3 |
| **Services for prevention and chronic diseases** | **2** | 4 |
| Patients asked about medical history and habits | 1 | 5 |
| Patient told when to come back for checkup | 2 | 4 |
| Patients weighed | 2 | 4 |
| Regular blood pressure checks for adults over 40 | 2 | 4 |
| Accurate treatment of adults with hypertension | 1 | 3 |

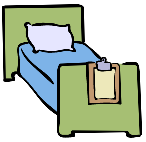

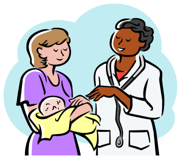

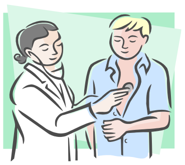

This information was prepared in January 2014 for your clinic by NAME.
If you have questions or comments please call NAME on 123 435 3142.
