# Supplementary material for: Developing citizen report cards for primary health care in low and middle-income countries: Results from cognitive interviews in rural Tajikistan
Source: PLoS One. 2017 Oct 24;12(10):e0186745. doi: 10.1371/journal.pone.0186745 (PMC5655492; doi:10.1371/journal.pone.0186745)
Supplement: S2 Fig — (PDF) [file pone.0186745.s002.pdf]

S2 Fig. Revised report card template.

How does your clinic compare?

This information helps you assess health care services provided by your local clinic, compared to the regional average.

Your clinic has a grade of 2 (unsatisfactory) which is <u>worse</u> than the regional average of 3 (satisfactory). Clinics are graded on a scale from 1 (bad) to 5 (excellent).

Your clinic: [NAME]

Higher numbers are better.

Compare with similar clinics

Average for this category

	Your clinic	Regional average
**<u>Overall grade</u>**	**<u>2</u>**	<u>3</u>
**General conditions**	**2**	3
**Cleanliness and medical equipment/drugs**<br>Clinic is clean and has basic medical equipment and drugs	3	4
**Respectful and responsive to patients**<br>Clinic treats patients with respect, explains costs before treatment and explains diagnosis	2	4
**Clinic management**<br>Clinic has regular opening hours and training	2	2
**Good condition of building**	1	3
**Services for mothers and children**	**3**	2
Children vaccinated by age 1	5	5
Children under age 5 monitored	4	5
Women with 1 checkup early in pregnancy	3	2
Women with 4 or more checkups during pregnancy	4	3
Accurate treatment of children's illnesses	4	3
**Services for prevention and treatment of chronic diseases**	**2**	4
Patients asked about medical history and habits	1	5
Patient told when to come back for checkup	2	4
Patients weighed	2	4
Regular blood pressure checks for adults over 40	2	4
Accurate treatment of adults with hypertension	1	3

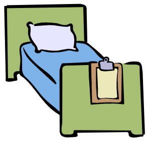

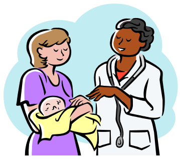

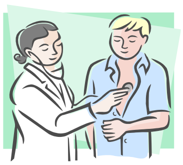

This information was prepared in January 2014 for your clinic by NAME.
If you have questions or comments please call NAME on 123 435 3142.
