# Supplementary material for: Developing citizen report cards for primary health care in low and middle-income countries: Results from cognitive interviews in rural Tajikistan
Source: PLoS One. 2017 Oct 24;12(10):e0186745. doi: 10.1371/journal.pone.0186745 (PMC5655492; doi:10.1371/journal.pone.0186745)
Supplement: S1 Table — (PDF) [file pone.0186745.s003.pdf]

S1 Table. Sample for cognitive interviews (counts).

Province	Gender	Age 18-45	Age 46+
Khatlon	Male	5	5
Khatlon	Female	5	5
Soghd	Male	5	5
Soghd	Female	5	5
Total	Male	10	10
Total	Female	10	10
